# Longer Telomere Length in Patients with Balkan Endemic Nephropathy Undergoing Chronic Hemodialysis Is Associated with Lower Cardiovascular Mortality

**DOI:** 10.34067/KID.0000000603

**Published:** 2024-10-02

**Authors:** Vedran Premužić, Simon Toupance, Allyson Hollander, Želimir Stipančić, Nikolina Bukal, Ana Jelaković, Ivan Brzić, Borna Čulig, Neda Slade, Athanase Benetos, Bojan Jelaković

**Affiliations:** 1 Department of Nephrology, Hypertension, Dialysis and Transplantation, University Hospital Center Zagreb, Zagreb, Croatia; 2 School of Medicine, University of Zagreb, Zagreb, Croatia; 3 Université de Lorraine, Inserm, DCAC, Nancy, France; 4 Department of Dialysis Odžak, County Hospital Orašje, Odžak, Bosnia and Herzegovina; 5 General Hospital Dr. Josip Benčević, Slavonski Brod, Croatia; 6 University of Rijeka, Rijeka, Croatia; 7 University Josip Juraj Strossmayer Osijek, Osijek, Croatia; 8 The Ruder Boskovic Institute, Zagreb, Croatia; 9 Université de Lorraine, CHRU-Nancy, Pôle “Maladies du Vieillissement, Gérontologie et Soins Palliatifs,” Nancy, France

**Keywords:** AKI, chronic dialysis, ESKD, hemodialysis, hypertension, pulse wave velocity

## Abstract

**Key Points:**

- Longer telomeres are associated with less cardiovascular mortality in patients undergoing chronic hemodialysis.
- In patients with Balkan endemic nephropathy, telomere length was negatively associated with arterial stiffness and positively associated with survival.
- The association of Balkan endemic nephropathy with slower vascular aging and longer telomere length may partially explain this phenomenon.

**Background:**

Balkan endemic nephropathy (BEN) is characterized with later onset and milder forms of hypertension and lower pulse wave velocity than other ESKD. Longer telomeres are associated with better cardiovascular (CV) prognosis. Therefore, we hypothesized that telomere length (TL) could be longer in patients with BEN compared with other patients with ESKD.

**Methods:**

A total of 124 patients undergoing hemodialysis (68 BEN, 56 non-BEN) were enrolled and followed-up for 72 months. TL was measured in leukocytes by Southern blot at inclusion.

**Results:**

Age- and sex-adjusted TL was significantly longer in the BEN group (*P* < 0.001). TL was negatively associated with carotid-femoral pulse wave velocity in patients with BEN. Patients with BEN had significantly lower CV mortality than patients with non-BEN ESKD (*P* < 0.001). In the BEN group, shorter TL (1 kb change) was the only determinant of shorter survival (hazard ratio, 0.11). Using the TL threshold defined by receiver operating characteristic analysis (TL <6.21 kb), we showed in both groups significantly higher CV mortality in the presence of short telomeres (log-rank [Mantel-*P* < 0.001]).

**Conclusions:**

Longer telomeres are associated with less CV mortality in patients undergoing chronic hemodialysis. Patients with BEN had longer TL and longer survival than that of patients with other ESKD. In patients with BEN, TL was negatively associated with arterial stiffness and positively associated with survival. This study confirmed our hypothesis that BEN is associated with slower vascular aging and that longer TL may partially explain this phenomenon.

## Visual Abstract

**Figure:**
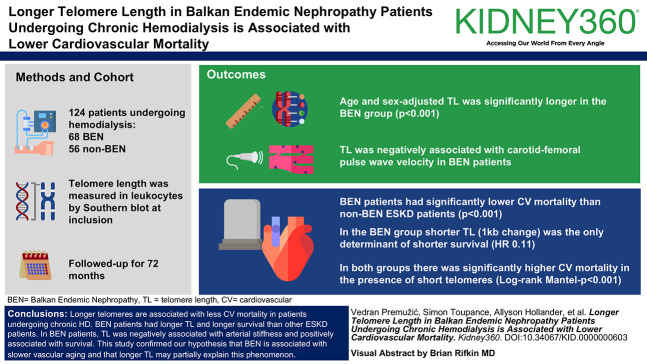

## Introduction

CKD is associated with increased cardiovascular (CV) morbidity and mortality, and it was assumed that CKD represents a state of accelerated or early vascular aging.^1^ Results on higher pulse wave velocity (PWV) in patients with CKD than in non-CKD population is in line with this observation.^2^ Patients with shorter telomeres show earlier and more severe CV and metabolic alterations, such as arterial stiffness, atherosclerotic CV disease, increased mortality, and shorter life expectancy.^3–12^ On the opposite, long telomeres are associated with an increased risk of several types of cancers and other proliferative diseases.^13^ Recent clinical longitudinal studies and genetic analyses using the Mendelian randomization approach showed that short telomeres preceded these clinical manifestations, indicating a causal role of telomeres on several age-related diseases.^7,12–14^ Telomere length (TL) could modulate the propensity for developing CV complications, especially in people at high CV risk, such as people with CKD.^7,15^

Balkan endemic nephropathy (BEN), an environmental form of worldwide presented aristolochic acid nephropathy, is a peculiar chronic tubulointerstitial nephropathy frequently associated with urothelial cancers of the upper urinary tract.^16–19^ BEN was considered a very slowly progressive CKD unaccompanied by hypertension, and the clinical course was characterized by normal BP until the uremic phase of ESKD.^16,19^ Recently, we reported on lower PWV in patients with BEN undergoing chronic hemodialysis compared with non-BEN patients despite being older.^20^ In the follow-up period of 25 months, there were significantly less CV deaths in patients with BEN than in other patients with ESKD undergoing dialysis, and we proposed that shorter duration of hypertension and milder forms of hypertension mostly contributed to the slower vascular aging in patients with BEN.

Given that patients with BEN have slower vascular aging, that is, lower PWV and later onset of hypertension that is milder compared with patients with other ESKD, we hypothesized that TL would be longer in patients with BEN and associated with lower PWV and less CV mortality.

## Methods

### Patients

In this prospective, longitudinal, multicentric study, 124 patients undergoing chronic hemodialysis were recruited from three dialytic centers (General Hospital Dr. Josip Benčević, Slavonski Brod, Croatia; General Hospital Odžak, Bosnia and Herzegovina; and University Hospital Center Zagreb, Croatia). Inclusion criteria were chronic hemodialysis for at least 3 months and signed informed consent. Exclusion criteria were atrial fibrillation or other chronic arrhythmias, stroke or myocardial infarction (in the 3 previous months), congestive heart failure New York Heart Association, III–IV, and significant hemodynamic instability during the dialysis, defined as at least one hypotensive episode during dialysis session per week. Protocol was approved by the Ethical Committees of institutions according to the Declaration of Helsinki and its subsequent modifications, and data were managed in accordance with the patients' written informed consent. This study was performed as a part of the research project of the Ministry of Science, Education and Sports of the Republic of Croatia “Endemic nephropathy in Croatia—epidemiology, etiology, and pathophysiology,” number: 108-00000000-0329. Diagnosis and classification were done according to the modified World Health Organization criteria for BEN.^16,19^ At baseline, BEN was diagnosed in 68 patients (30 men, 38 women) and in 56 patients, ESKD was caused by other diseases (non-BEN) (30 men, 26 women). At the end of follow-up, 52 patients with BEN (22 men, 30 women) and 34 non-BEN patients (18 men, 16 women) were alive and eligible for analyses. In the whole group, the main cause of ESKD was BEN (54.8%). In the non-BEN group, the most frequent causes of ESKD were diabetes, hypertension, chronic GN, and autosomal dominant polycystic kidney disease (28%, 23.5%, 14.2%, and 7.2%, respectively). All patients were studied on the mid-week dialysis day after a 2-day interdialytic interval.

### Clinical Assessments

Body mass index was calculated as the dry weight divided by the square of body height. Brachial BP was measured before the dialytic session using the Omron M6 device (Kyoto, Japan) in a sitting position on the nonfistula arm. Carotid-femoral PWV and aortic augmentation index (AIx) were assessed by Tensiomed Arteriograph (Medexpert Ltd., Budapest, Hungary), a computerized device using an oscillometric method which simultaneously measure brachial BP, PWV, and AIx. PWV and AIx were determined as a mean of three measurements on the nonfistula arm. All measurements were done by the same physician (V. Premužić). Data on medical history and drug therapy were collected from medical records. We used the Cornell electrocardiography criteria for diagnosing left ventricular hypertrophy.

### TL Measurements by Southern Blot

Leukocyte DNA was extracted from frozen buffy coats obtained at inclusion with a salting out protocol (Puregene kit, Qiagen, Hilden, Germany) and resuspended in tris and EDTA 1× buffer. DNA quantity was assessed by spectrophotometry (Nanodrop, Thermo Fisher Scientific, Waltham, MA). DNA samples passed an integrity test using a 1% (wt/vol) agarose gel before TL measurement was performed by the Southern blot analysis of terminal restriction fragments, as described previously.^21^ Briefly, DNA samples were treated overnight with restriction enzymes HinfI and RsaI (Roche Diagnostics GmbH, Mannheim, Germany). Digested DNA samples and DNA ladder were resolved on 0.5% (wt/vol) agarose gels for 23 hours. After depurination, denaturation, and neutralization, DNA was transferred on a positively charged nylon membrane (Roche Diagnostics GmbH) using a vacuum blotter (Biorad, Hercules, CA). Membranes were hybridized at 42°C with a digoxigenin (DIG)-labeled telomeric probe and a DIG-labeled ladders probe. The probes were later detected by the DIG luminescent detection procedure (Roche Diagnostics GmbH) and exposed on a charge-coupled device camera (Las 4000, Fujifilm Life Sciences, Cambridge, MA). Measurements were performed in duplicate on separate membranes. The interassay coefficient of variation for the duplicate measurements was below 2%.

### Statistical Analyses

Statistical analyses were performed using SPSS version 23.0 (IBM Corp.). Normality of data distribution was tested using the Kolmogorov–Smirnov test. Categorical data were expressed as numbers and frequencies. Correlations were obtained using the Pearson test for normally distributed variables and Spearman rank correlation for non-normally distributed variables. Normally distributed variables were presented as means±SDs and the Student *t* test for independent samples was used for comparisons between two groups. Non-normally distributed data were presented as median and interquartile range, and the Mann–Whitney *U*-test was used in comparison between two groups. Baseline-to-follow-up comparisons were done using the Student *t* test for paired samples and the Wilcoxon test. *Post hoc* Tukey–Kramer test was done before and after adjustment for age and sex. Categorical variables were compared using the chi-squared test. Multiple linear regression and multiple nominal regression were used to explore the influence of different variables on TL and PWV, whereas logistic regression was used for categorical dependent variables. A *P* value < 0.05 (two-sided tests) was considered significant. Survival probability curves were generated by means of the Kaplan–Meier method and analyzed by the log-rank (Mantel–Cox) test. Hazard ratios (HRs) and 95% confidence intervals (CIs) were estimated by the Cox proportional hazards regression method (Cox regression). Survival was defined as the time interval from the patient enrollment until the date of death, which was the point when patients were censored. To evaluate the sensitivity and specificity of TL, a receiver operating characteristic (ROC) curve analysis was performed.

## Results

### Clinical Characteristics of Patients With BEN and Non-BEN ESKD

**Figure 1:**
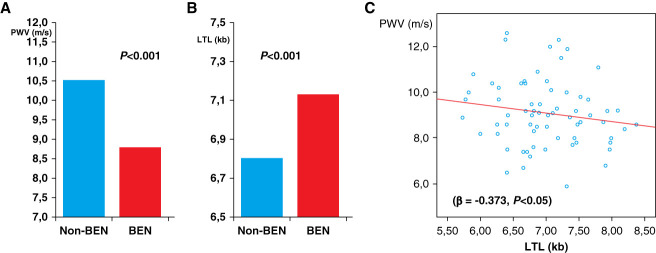
**Arterial stiffness measured by carotid-femoral PWV and TL in patients with BEN and non-BEN patients.** (A) Age- and sex-adjusted PWV in patients with BEN and non-BEN patients. Values are expressed as mean. (B) Age- and sex-adjusted leukocyte TL in patients with BEN and non-BEN patients. Values are expressed as mean. (C) Negative relation between leukocyte TL and PWV in the BEN group. BEN, Balkan endemic nephropathy; kb, kilobase; LTL, leukocyte telomere length; PWV, pulse wave velocity; TL, telomere length.

Demographic, clinical, and laboratory data of enrolled patients are shown in Tables 1 and 2. Patients with BEN were significantly older than non-BEN patients (*P* < 0.001). They presented substantially less diabetes (5.9% versus 10.7%) and left ventricular hypertrophy (42.6% versus 51.8%) compared with the non-BEN group. Ultrafiltration was significantly higher, while duration of dialysis was significantly shorter in the non-BEN than in the BEN group. There was no difference in the prevalence of hypertension between the two groups, but duration of hypertension prior the start of hemodialysis was significantly shorter in patients with BEN than in non-BEN patients. Patients with BEN were treated with significantly smaller number of antihypertensive drugs. At baseline, brachial systolic BP, diastolic BP, and mean arterial pressure were higher in patients with BEN, but there was no difference between the two groups in brachial pulse pressure (PP) and central systolic BP. At the end of follow-up, there was no difference in any BP value between the two groups (Table 3). PWV was significantly lower in patients with BEN at baseline (*P* < 0.004) and at the end of follow-up (*P* < 0.01), even before adjusting for age, and this is even stronger after adjusting for age (both *P* < 0.005, Figure 1A). There were significantly more upper urothelial cancers in the BEN than in the non-BEN group (*P* < 0.001).

**Table 1:** Basic demographic and clinical data of entire group and subgroups of patients according to the BEN status

Demographic and Clinical Data	Entire Cohort (*n*=124)	Non-BEN (*n*=56)	BEN (*n*=68)	*P* Value	*P* Value^a^
Age baseline (yr)	65±14	57±16	72±6	<0.001	
Age at the end of follow-up (yr)	67±14	59±16	73±6	<0.001	
Age on start of hemodialysis	58±16	49±18	66±8	<0.001	—
Height (kg)	167±9	169±9	166±8	0.04	0.74
Weight (cm)	66±14	69±15	64±12	0.03	0.02
BMI (kg/m^2^)	24±4	25±4	23±4	0.06	0.003
Sex (% men)	48.4	53.6	44.1	0.30	—
Waist circumference (cm)	80±11	83±12	78±10	0.01	0.01
Smokers (%)	12.9	12.5	13.2	0.90	0.03
Hypertension (%)	89.5	89.3	89.7	0.94	0.15
Diabetes type 2 (%)	8.1	10.7	5.9	0.33	0.03
Upper tract urothelial cancer (%)	7.2	0	13.2	<0.001	<0.001
Other cancers (%)	4.8	10.7	0	<0.01	<0.01
LVH (%)	46.8	51.8	42.6	0.32	
Duration of hypertension prior to hemodialysis (mo)	72 (43–134)	86 (52–145)	60 (38–102)	0.10	0.009
Antihypertensive therapy (yes, %)	78.2	87.5	70.6	0.02	0.005
RAAS blockers (%)	37.3	35.1	40.2	0.42	0.22
Calcium channel blockers (%)	60.5	55.4	64.7	0.29	0.09
*β* blockers (%)	33.1	46.4	22.1	0.004	0.006
Diuretics (%)	29.8	50.0	13.2	<0.001	<0.001
*α* blockers (%)	16.1	32.1	2.9	<0.001	<0.001
Moxonidine (%)	13.7	23.2	5.9	0.005	0.054
Vasodilators (%)	4.8	10.7	0.0	0.005	0.04
Months on dialysis	78 (34–112)	88 (45–129)	70 (31–103)	0.08	0.13
Vascular access fistula (%)	84.7	82.1	86.8	0.48	0.19
Duration of dialysis (hours)	4.26±0.61	3.95±0.48	4.51±0.59	<0.001	<0.001
Ultrafiltration (ml)	3452±700	3661±549	3279±765	0.002	0.02
Residual diuresis (ml)	271 (110–482)	252 (102–467)	286 (128–532)	0.62	0.82
HCV positive (%)	4.0	7.1	1.5	0.11	0.77
HBV positive (%)	0.8	0.0	1.5	0.37	0.19
Vitamin D weekly dose (mg)	0.92 (0.24–1.41)	0.95 (0.26–1.44)	0.90 (0.22–1.38)	0.73	0.57
Phosphate binders dose (g)	2.17 (1.31–2.92)	2.29 (1.34–3.01)	2.07 (1.22–2.86)	0.56	0.95
EPO weekly dose (IU)	8167±3420	8085±3209	8224±3584	0.83	0.43
TL (kb)	6.95±0.69	6.89±0.76	6.99±0.63	0.42	0.0009
Died (%)	30.6	39.3	23.5	0.06	<0.001
Survival months	59±21	55±24	63±18	0.03	<0.001

Values are expressed as mean±SD or %. BEN, Balkan endemic nephropathy; BMI, body mass index; EPO, erythropoietin; HBV, hepatitis B virus; HCV, hepatitis C virus; kb, kilobase; LVH, left ventricular hypertrophy; RAAS, renin-angiotensin-aldosterone system; TL, telomere length.

a Adjusted for age and sex.

**Table 2:** Laboratory data of entire group and subgroups according to the BEN status at baseline and at the end of follow-up period

Laboratory Data	Entire Cohort (*n*=124)	Non-BEN (*n*=56)	BEN (*n*=68)	*P* Value	*P* Value^a^
Baseline					
Serum creatinine (*µ*mol/L)	728±214	735±184	721±238	0.72	0.23
Hb (g/L)	113±14	106±12	118±14	<0.0001	<0.001
Potassium (mmol/L)	5.08±0.80	5.04±0.74	5.12±0.86	0.57	0.22
Sodium (mmol/L)	139±3	139±3	139±3	0.12	0.29
Calcium (mmol/L)	2.16±0.37	2.23±0.43	2.10±0.31	0.053	0.09
Phosphate (mmol/L)	1.45±0.55	1.63±0.56	1.31±0.50	0.001	0.07
iPTH (pmol/L)	26±32	31±33	22±31	0.15	0.38
Fasting blood glucose (mmol/L)	5.81±1.76	5.86±2.24	5.78±1.26	0.81	0.33
Total cholesterol (mmol/L)	4.74±0.99	4.70±0.89	4.78±1.08	0.65	0.049
Triglycerides (mmol/L)	2.05±1.08	1.96±0.47	2.12±1.40	0.43	0.33
Uric acid (mmol/L)	326±51	323±53	328±49	0.61	0.15
**End of follow-up period**					
Serum creatinine (*µ*mol/L)	789±224	792±202	741±198	0.55	0.21
Hb (g/L)	112±13	107±12	117±14	<0.001	<0.001
Potassium (mmol/L)	5.12±0.81	5.05±0.76	5.14±0.87	0.56	0.23
Sodium (mmol/L)	139±3	139±3	139±3	0.24	0.33
Calcium (mmol/L)	2.12±0.34	2.19±0.40	2.10±0.31	0.06	0.09
Phosphate (mmol/L)	1.53±0.59	1.60±0.62	1.41±0.52	0.04	0.08
iPTH (pmol/L)	32±29	35±33	25±30	0.21	0.44
Fasting blood glucose (mmol/L)	5.82±1.64	5.94±2.35	5.78±2.14	0.77	0.39
Total cholesterol (mmol/L)	4.44±0.92	4.31±0.81	4.52±1.12	0.43	0.06
Triglycerides (mmol/L)	2.14±1.31	1.89±0.35	2.19±1.41	0.30	0.22
Uric acid (mmol/L)	367±57	373±59	349±50	0.47	0.10

Values are expressed as mean±SD or %. BEN, Balkan endemic nephropathy; Hb, hemoglobin; iPTH, intact parathormone.

a Adjusted for age and sex.

**Table 3:** Hemodynamic characteristics of non-BEN patients and patients with BEN at baseline and at the end of follow-up period

Hemodynamic Characteristics	Entire Cohort (*n*=124)	Non-BEN (*n*=56)	BEN (*n*=68)	*P* Value	*P* Value^a^
**Baseline**					
Brachial systolic BP (mm Hg)	157±33	152±33	162±32	0.10	0.03
Brachial diastolic BP (mm Hg)	87±17	85±19	88±15	0.34	0.03
Brachial PP (mm Hg)	71±21	67±22	74±20	0.09	0.13
Brachial mean BP (mm Hg)	110±21	107±22	113±20	0.18	0.02
HR (beats/min)	73±13	73±12	74±13	0.54	0.06
Central systolic BP (mm Hg)	159±37	154±37	163±37	0.14	0.11
PWV (m/s)	9.6±1.8	10.2±2.0	9.1±1.5	0.004	<0.001
AIx (%)	38±17	37±16	39±17	0.62	0.15
**End of follow-up period**					
Brachial systolic BP (mm Hg)	157±28	158±26	157±30	0.87	0.98
Brachial diastolic BP (mm Hg)	85±15	85±15	85±16	0.96	0.30
Brachial PP (mm Hg)	72±20	73±19	72±21	0.84	0.41
Brachial mean BP (mm Hg)	111±20	111±19	111±20	0.99	0.60
HR (beats/min)	74±12	74±12	74±13	0.84	0.60
Central systolic BP (mm Hg)	161±33	162±33	160±33	0.68	0.70
PWV (m/s)	9.9±1.7	10.4±1.8	9.6±1.6	0.01	0.005
AIx (%)	42±15	42±16	42±14	0.93	0.48

Results are shown as mean±SD or median (interquartile range). AIx, augmentation index; BEN, Balkan endemic nephropathy; HR, heart rate; PP, pulse pressure; PWV, pulse wave velocity.

a Adjusted for age and sex.

### TL According to BEN Diagnosis

The average TL in the entire cohort was 6.95±0.69 kb. There was no difference in TL between men and women (6.92±0.75 kb versus 6.98±0.61 kb; *P* > 0.05). No difference in unadjusted TL was observed between patients with BEN and non-BEN patients; however, after adjustment for age and sex, TL was significantly longer in the BEN group (Figure 1B). In the entire population, as expected, TL was negatively associated with age (Supplemental Figure 1A). When analyzed separately, this relationship was observed in the non-BEN group only (*β*=−0.680, *P* < 0.01, Supplemental Figure 1B). In the BEN group, TL was negatively associated with PWV (*β*=−0.373, *P* < 0.05, Figure 1C), but such relationship was not observed in the non-BEN group. In univariate analyses, TL was negatively associated with duration of hypertension before the start of chronic hemodialysis in the entire group and in non-BEN patients. In multivariate analysis, duration of hypertension prior starting dialysis was not associated with TL in patients undergoing chronic hemodialysis. In multinominal regression analysis, we found that only diagnosis of BEN was significantly associated with TL. On logistic regression, older age and longer TL were independently associated with the diagnosis of BEN (odds ratio, 1.12 [95% CI, 1.07 to 1.18]; odds ratio, 2.32 [95% CI, 1.15 to 4.65], respectively). TL was significantly longer in patients with BEN with upper urothelial cancer than in non-BEN patients with other cancers (7.01±0.32 versus 6.5±0.22; *P* = 0.02).

### Mortality Data According to BEN Diagnosis and TL

**Figure 2:**
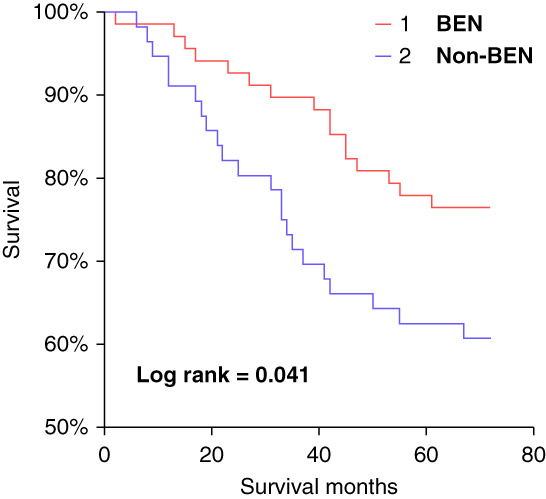
**Kaplan–Meier analysis of CV mortality between patients with BEN and non-BEN patients.** BEN—red line; non-BEN—blue line. CV, cardiovascular.

At the end of follow-up, the mortality rate in the entire cohort was 30.6%, being significantly higher in the non-BEN group (Table 1). Patients with BEN died significantly less frequently from CV events than non-BEN patients (*P* < 0.001). In non-BEN patients, stroke, myocardial infarction, and heart failure were causes of CV deaths in 32.5%, 27.2%, and 40.3% cases, respectively. In patients with BEN, the respective mortality rates were 17.7%, 49.2%, and 33.1%, respectively. Kaplan–Meier curves and log-rank test showed significantly lower CV mortality in the BEN group (63.2 [95% CI, 59.0 to 67.5] versus 54.9 [95% CI, 48.7 to 61.0] months; log-rank *P* = 0.04) (Figure 2). Patients who died from CV events had significantly shorter TL, both in the BEN and in the non-BEN group (6.42±0.44 versus 7.16±0.56; *P* < 0.001; 6.56±0.71 versus 7.10±0.73; *P* = 0.008, respectively). On univariate analysis, TL was correlated with survival in the entire cohort (*r*=0.387; *P* = 0.001). In the linear regression model, TL was the strongest predictor of longer survival (*β*=0.419, *P* < 0.05) in the BEN group. No such association was found in the non-BEN group. The Cox regression analysis in the entire population (Table 4) showed that older age, diagnosis of non-BEN, and shorter TL were determinants of CV mortality. Older age in the non-BEN group and shorter TL in the BEN group were the only determinants of CV mortality (Table 4).

**Table 4:** Determinants of survival in the entire population and in the two subgroups according to age, sex, and TL

Independent Variable	Regression Coefficient (B)	SEM of B	Risk Ratio Exp (B)	Lower 95.0% C.L. of Exp (B)	Upper 95.0% C.L. of Exp (B)	Mean	Wald Z-Value	Prob Level	Pseudo R^2^
**Entire cohort**									
Age (1 yr)	0.066363	0.020652	1.0686	1.0262	1.1128	65.0968	3.2134	0.0013	0.2268
Diagnosis of BEN	−1.184059	0.379493	0.306	0.1455	0.6439	0.5484	−3.1201	0.0018	0.2167
Sex	0.397932	0.343251	1.4887	0.7597	2.9174	0.4839	1.1593	0.2463	0.0368
TL (1 kb)	−0.936766	0.268776	0.3919	0.2314	0.6637	6.9474	−3.4853	0.0005	0.2566
**Non-BEN group**									
Age	0.080768	0.022022	1.0841	1.0383	1.1319	57.2857	3.6675	0.0002	0.4086
Sex	0.773401	0.466982	2.1671	0.8677	5.4123	0.5357	1.6562	0.0977	0.1235
TL	−0.374482	0.298046	0.6876	0.3834	1.2333	6.8916	−1.2565	0.2089	0.075
**BEN group**									
Age	0.050738	0.047787	1.052	1.052	0.958	1.1553	1.0618	0.2883	0.0759
Sex	0.186235	0.502707	1.2047	1.2047	0.4498	3.2269	0.3705	0.711	0.0099
TL	−2.136624	0.568002	0.1181	0.1181	0.0388	0.3594	−3.7616	0.0002	0.5077

BEN, Balkan endemic nephropathy; C.I., confidence intervals; Exp, exponential; Prob, probability; TL, telomere length.

**Figure 3:**
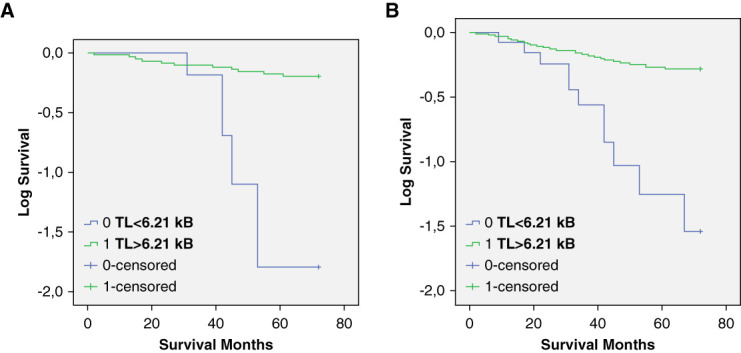
**Kaplan–Meier analysis of survival probability according to short versus long telomere.** Entire cohort (A) and BEN group (B) of patients' survival curves for CV mortality.

ROC analysis in the BEN group of patients revealed TL was useful for prediction of CV mortality, as the area under the curve was 75.5% (Supplemental Figure 2). A cutoff point for TL, based on the maximum sensitivity (96%) and highest specificity (71%), was determined at 6.21 kb. Figure 3, A and B shows Kaplan–Meier analysis of survival probability according to short versus long TL (below and above 6.21 kb) in the entire cohort and in patients with BEN. A log-rank (Mantel–Cox) test showed significant differences in mortality between patients with short and long TL, both in the entire cohort (Figure 3A) and in the BEN group (Figure 3B) (*P* < 0.001 for both). The impact of TL on CV mortality was studied with the Cox proportional HR model, considering confounding factors in a stepwise manner (Supplemental Table 1). In a crude analysis, a TL <6.21 kb revealed a HR of 4.34 (95% CI, 1.18 to 6.42), *P* < 0.001. HR for TL independently contributed to patient survival after additional adjustments for age, sex, and PWV (HR, 5.45 [95% CI, 2.34 to 9.12]; *P* < 0.001). In the entire cohort, we found significant difference in survival in men with short versus long TL (*P* < 0.001) and in women with short versus long TL (*P* = 0.03) (Supplemental Figure 3, A and B).

## Discussion

The main findings of our study are (*1*) TL was significantly longer in patients with BEN compared with patients with other ESKD and (*2*) TL was an important predictor of CV mortality in patients undergoing chronic hemodialysis.

Average TL in our entire cohort is in concordance with reports of other authors on TL in patients undergoing chronic hemodialysis.^21,22^ We failed to find an association of TL with dialysis vintage. This contrasts with results of other authors who reported that longer duration of hemodialysis is associated with shorter TL.^23^ However, our results are in line with data published by Carrero *et al.* who found no association of dialysis vintage and TL.^22^ Wang *et al.* found association of dialysis vintage and TL only in the age group 53–74 years, but not in younger patients.^24^ In our population, there were no difference in TL between men and women after adjusting for age, which is probably due to the relatively small number of individuals. In univariate analyses, TL was negatively associated with duration of hypertension before the start of chronic hemodialysis in the entire group and in non-BEN patients, which is line with data published by Tellechea and Pirola.^25^ The lack of this association in patients with BEN is one of the possible reasons we have not found an association of age and TL in this subgroup. The Framingham Heart Study has identified an association between hypertension and short TL.^26^ TL has been reported to be inversely associated with increased systolic BP and PP.^25,27^ Yu *et al.* observed that longer TL are protective for hypertension, and results of meta-analyses conducted by Tellechea and Pirola confirmed that leukocyte TL may be shorter in hypertensive than in normotensive individuals.^25,27^ However, in multivariate analysis, duration of hypertension before starting dialysis was not associated with TL in patients undergoing chronic hemodialysis. In multinominal regression analysis, we found that only diagnosis of BEN was significantly associated with TL.

Age- and sex-adjusted TL was significantly longer in patients with BEN than in non-BEN patients. Longer TL in patients with BEN could explain later onset and milder forms of CV diseases because it has been demonstrated, in non-CKD patients, that longer TL is protective for CV diseases.^15^ We found difference in associations of TL between patients with BEN and non-BEN patients undergoing chronic hemodialysis. In the BEN group, TL is negatively associated with PWV, that is, patients with shorter telomeres have increased risk for developing arterial stiffness.

Searching literature, we failed to find any report on TL and PWV in patients undergoing chronic hemodialysis. In a group of untreated patients with high-normal BP, we have found that in men, but not in women, TL significantly contributed to PP and PWV.^3^ Raymond *et al.* found that sex and premenopausal status do not affect age-related decrease in TL and the association between TL and PWV.^28^ McDonell *et al.* analyzed association of TL and PWV in healthy individuals and reported that in patients younger than 30 years, TL was significantly shorter in those with high PWV, whereas in patients older than 50 years, TL was significantly longer in those with high PWV.^4^ In a group of patients with increased CV risk, Eguchi *et al.* did not find convincing association of TL with CV measures of arterial stiffness and target organ damage.^29^ Our study is the first report on association between TL and PWV in patients undergoing chronic hemodialysis. Observed difference in association of TL and PWV between patients with BEN and non-BEN patients could reflect differences in exposure to some risk factors. Raschenberger *et al.* proposed that circumstances that lead to CKD development or are associated with CKD, such as activity of renin-angiotensin system, oxidative stress, or higher expression of p53, might have strong impact on TL.^30^ The results of this study are suggesting that BEN is associated with slower vascular aging where longer telomeres may partially explain this phenomenon. A later onset and milder forms of hypertension in patients with BEN during predialytic and dialytic clinical course most often explained by loss of sodium because of tubulopathy, which also occurs in other forms of interstitial nephritis, can be an additional explanation.^31^ Damage to juxtaglomerular cells and proximal tubules at the beginning of the disease probably leads to reduced synthesis of renin and angiotensin converting enzyme, which can contribute to normotension and, additionally, explain the later appearance of hypertension in BEN. Likewise, the medulla of the kidney is spared in the early stages of the disease and its function is preserved, which includes synthesis of PG, PGE2, and PGI2. There are several studies that describe renal proximal tubule damage as an early manifestation toxicity of aristolochic acid.^32,33^

Association of leukocyte TL with mortality among general adult population was reported in many, but not in all, studies.^5,6,34^ The report from the German CKD study conducted in a large cohort of patients with moderate CKD has been the only study on association of TL with CV mortality in CKD so far.^35^ Their results supported an association of TL with CV mortality. Our study is the first report on association of TL and CV mortality in patients with ESKD undergoing chronic hemodialysis. TL was significantly shorter in patients who died compared with survivors. To analyze the diagnostic value of TL for CV mortality, we used ROC analysis that revealed TL useful for prediction of CV mortality. A cutoff point for TL was determined at 6.21 kb what is in concordance with the cutoff point of 6.28 kb identified by Carrero *et al.* in their study on the role of short TL in all-cause mortality in patients undergoing chronic hemodialysis.^22^ In our study, TL independently contributed to patient survival after additional adjustments for age, sex, and PWV. On the contrary, Stefanidis *et al.* observed in their small group of patients on hemodialysis that only age and not TL was an independent survival predictor.^36^ This difference could be explained by the greater number of patients enrolled in our study. CV mortality in the BEN group was significantly lower than in the non-BEN group, and we observed significantly longer survival in the BEN group. TL was significantly shorter in the subgroup of patients who died compared with the subgroup of survivors, both in patients with BEN and in non-BEN patients. We found significant differences in survival between patients with short and long TL both in the BEN group and in the non-BEN group. On contrary, Stefanidis *et al.* observed in their group of patients on hemodialysis that only age and not TL was an independent survival predictor.^21^ In our entire cohort, increased age, shorter TL, and non-BEN diagnosis were associated with increased CV mortality. Interestingly, when analyzing predictors of CV mortality separately in our two subgroups, we found that in non-BEN patients only age and in patients with BEN only TL were independently associated with increased CV mortality. Observed difference between patients with BEN and non-BEN patients is in line with suggestion of other authors that mixed associations might reflect, among others, the heterogeneous spectrum of CKD.^29,30,37,38^ The percentages of CV mortality in our study are similar as in other studies investigating CV mortality in the dialysis population at a similar follow-up time intervals.^39,40^

**Figure 4:**
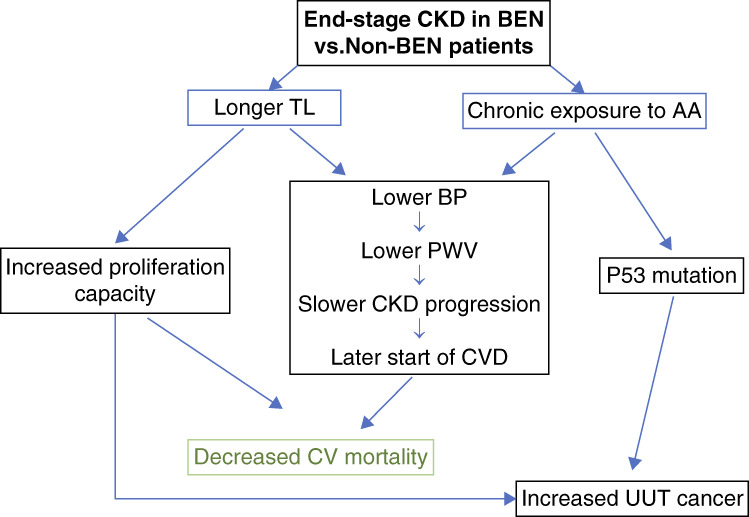
**Oversimplification of proposed mechanisms for the less CV mortality and more UUT cancers associated with longer TL in patients with BEN.** AA, aristolochic acid; CVD, cardiovascular disease; UUT, upper urothelial tract.

Recently, it was reported that longer leukocyte TL is associated with several cancers.^13,37^ This notion was supported by results from Mendelian randomized studies using leukocyte TL-associated single nucleotide polymorphisms, indicating that longer leukocyte TL has a causal relation to cancer risk.^13,37^ BEN is frequently associated with upper urothelial cancers, and in this study, we found that TL are longer in patients with BEN compared with other ESKD. Patients with BEN with upper urothelial cancer had significantly longer TL than patients with BEN without upper urothelial cancer, so it could be hypothesized that longer TL contribute to the increased upper urothelial cancer risk, usually related to the frequently presented characteristic TP53 mutation in BEN (Figure 4).^16,17,19,41–43^ Mendelian randomization studies also demonstrated that short telomeres have a causal role in the development of degenerative age-related diseases, such as arterial stiffness and renal damage.^14,15,28^ Therefore, we can propose that BEN CKD is a disease of exposure and that the balance between its complications, cardiovascular disease, and upper urothelial tract cancer is influenced by TL.

Our study has several limitations. TL was not measured at the start of dialysis. This would significantly improve understandings of TL biology during the chronic hemodialysis. Relatively small number of patients were recruited. However, our study is among the three largest studies on TL in patients undergoing chronic hemodialysis, and we compensate the modest sample size with the reproducibility of our measurements (https://trn.tulane.edu/resources/study-design-analysis/). BP was measured only in office, before hemodialysis session, and more accurate information would be obtained if we had used ambulatory blood pressure monitoring. Some could argue that we failed to find the association of TL with dialysis vintage because patients with BEN had shorter dialysis vintage. Although this can be one of the possible reasons, we must emphasize a previously reported slow progression of BEN to ESKD which could be prolonged with the appropriate treatment.^44–46^ The additional explanations regarding different dialysis vintage between groups is later onset and milder forms of hypertension during the predialytic clinical course of BEN.^45–47^ The two groups in this study are very heterogeneous and have many confounding factors and, therefore, statistical analyses and obtained results should be taken with caution.

Our study has several important strengths. This is the first report on association of TL with CV mortality in patients undergoing chronic hemodialysis. This is the first report on association of TL and PWV in patients undergoing chronic hemodialysis. Finally, this is the first report on association of TL with BEN.

Longer telomeres are associated with less CV mortality in patients undergoing chronic hemodialysis, regardless of the ESKD etiology. Patients with BEN had longer TL and longer survival than patients with other ESKD, and their TL was negatively associated with arterial stiffness and positively associated with survival. The present results suggest that BEN is associated more with slower vascular aging than other ESKD and that longer telomeres may partially explain this phenomenon. However, patients with BEN with upper urothelial cancer had longer telomeres than patients with BEN without cancer. We can propose that BEN CKD is a disease of exposure and that the balance between its complications, cardiovascular disease, and upper urothelial tract cancer is influenced by TL (Figure 4). Later onset and better control of hypertension, lower arterial stiffness, and, consequently, slower vascular aging and CV mortality in patients with BEN, which in this study was significantly associated with longer TL, can serve as a model and potential modifying risk factor of CV in other chronic hemodialysis patients.

## Supplementary Material

## Data Availability

All data are included in the manuscript and/or supporting information.

## Supplemental Material

Supplemental Figure 1. Correlation of telomere length with age in the entire population (A) and in each subgroup separately (B).

Supplemental Figure 2. Receiver-operating characteristic (ROC) curves analysis of the telomere length for predicting mortality.

Supplemental Table 1. Crude and adjusted relative risk of CV mortality in the entire group.

Supplemental Figure 3. Kaplan–Meier analysis of survival probability according to short versus long telomere in men (A) and in women (B).
