# Supplementary material for: Longer Telomere Length in Patients with Balkan Endemic Nephropathy Undergoing Chronic Hemodialysis Is Associated with Lower Cardiovascular Mortality
Source: Kidney360. 2024 Oct 2;5(12):1871–80. doi: 10.34067/KID.0000000603 (PMC11687986; doi:10.34067/KID.0000000603)
Supplement: Supplementary file 2 [file kidney360-5-1871-s002.pdf]

Supplementary materials

Table of contents:

Supplementary Figure S1 a-b. Correlation of telomere length with age in the entire population (a) and in each sub-group separately (b)

Supplementary Figure S2. Receiver-operating characteristic (ROC) curves analysis of the telomere length for predicting mortality

Supplementary Table S1. Crude and adjusted relative risk of a cardiovascular mortality in the entire group

Supplementary Figure S3 a-b. Kaplan-Meier analysis of survival probability according to short versus long telomere in men (a) and in women (b)

Telomere length vs. age in the entire population (left) and in each group separately

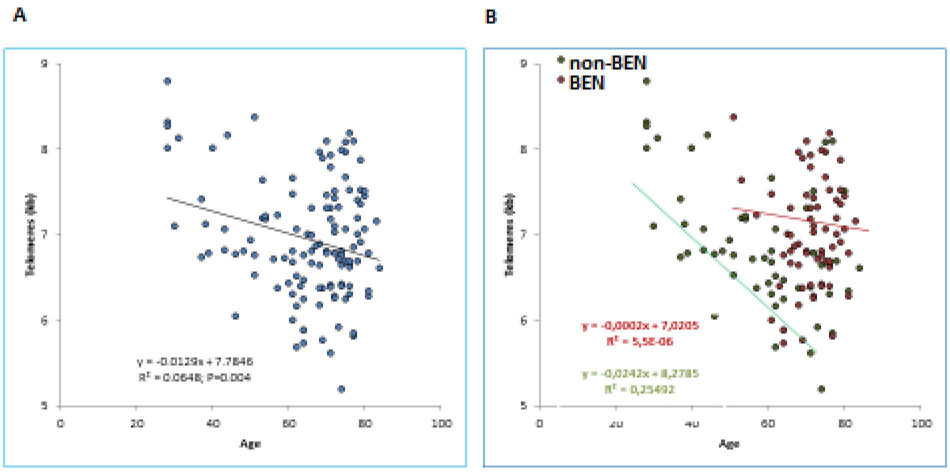

Supplementary Figure S1 a-b. Correlation of telomere length with age in the entire population (a) and in each sub-group separately (b)

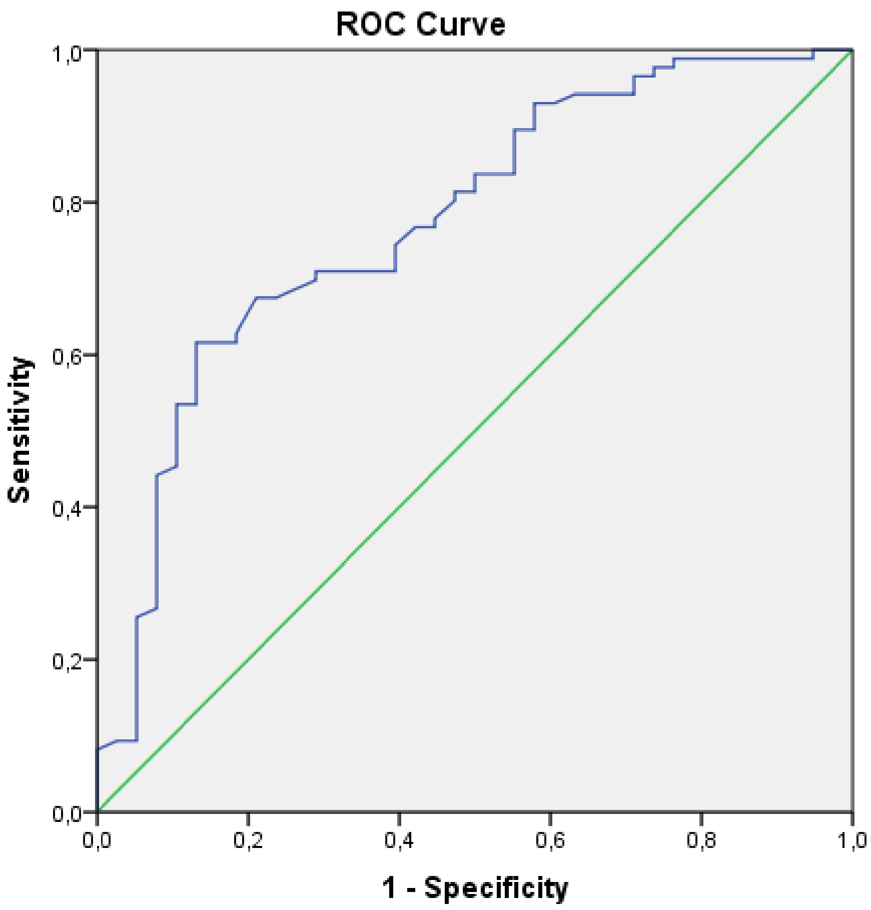

Supplementary Figure S2. Receiver-operating characteristic (ROC) curves analysis of the telomere length for predicting mortality in BEN group of patients

Supplementary Table S1. Crude and adjusted relative risk of a cardiovascular mortality in the entire group

Variables	Model 1			Model 2			Model 3			Model 4		
	HR	95% CI	p-value	HR	95% CI	p-value	HR	95% CI	p-value	HR	95% CI	p-value
TL (< 6.21 kb)	4.34	(1.18–6.42)	<0.001	4.78	(3.33-5.89)	<0.001	5.00	(2.55-7.31)	<0.001	5.45	(2.34-9.12)	<0.001
Age (< 65 years)				0.45	(0.26-0.68)	0.06	0.5	(0.32-0.71)	0.06	0.53	(0.18-0.88)	0.08
Gender (female)							1.58	(0.90-2.41)	0.17	1.61	(0.82-2.02)	0.15
PWV (< 10 m/s)										1.32	(1.02-1.52)	0.43

Data are presented as hazard ratios, 95% confidence intervals and P-values. Data adjustment for age (model 2), as well as gender (model 3) and pulse wave velocity (model 4). Cut-off points for telomere length was derived from receiver operating characteristic (ROC) curve analysis

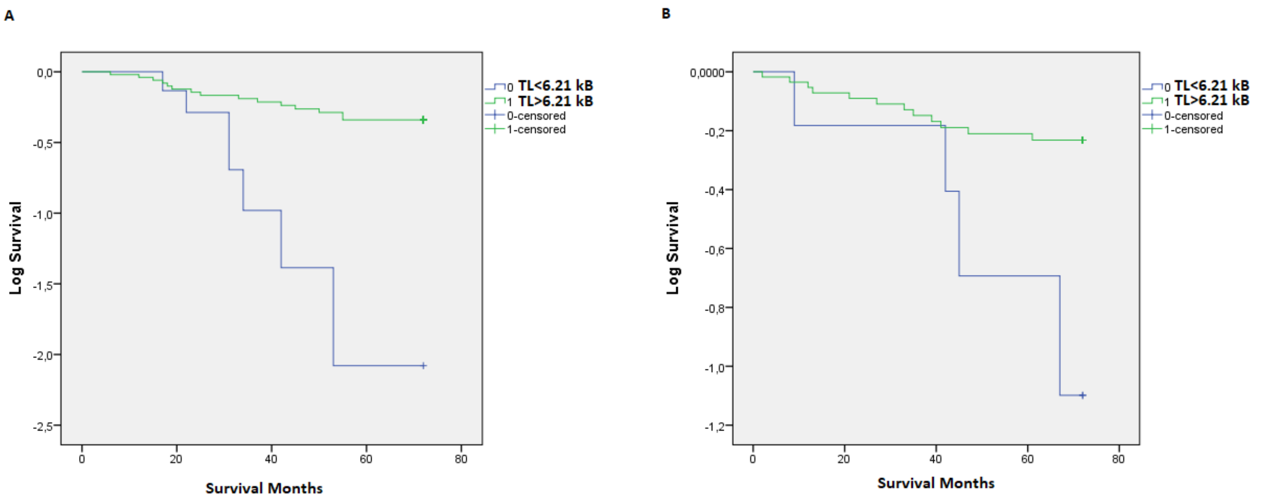

Supplementary Figure S3 a-b. Kaplan-Meier analysis of survival probability according to short versus long telomere in men (a) and in women (b)

Patients were stratified into two groups according to the cut-off point for TL as defined by ROCcurves (below and above 6.21 kb). On comparison, performed by log-rank (Mantel-Cox) test, there was significant difference in men ($P<0.001$) and women ($p=0.03$).
